# Hif-1α/Slit2 Mediates Vascular Smooth Muscle Cell Phenotypic Changes in Restenosis of Bypass Grafts

**DOI:** 10.1007/s12265-023-10384-8

**Published:** 2023-04-25

**Authors:** Sen Li, Zhiwei Gao, Haiqing Li, Chang Xu, Bing Chen, Qing Zha, Ke Yang, Weilin Wang

**Affiliations:** 1 Department of Vascular Surgery, The Second Affiliated Hospital, Zhejiang University School of Medicine, Hangzhou, Zhejiang 310009 China; 2 Department of Cardiovascular Medicine, Ruijin Hospital, Shanghai Jiaotong University School of Medicine, Shanghai, 200025 China; 3 Department of Hepatobiliary and Pancreatic Surgery, The Second Affiliated Hospital, Zhejiang University School of Medicine, Hangzhou, Zhejiang 310009 China; 4 Department of Cardiology, Shanghai Ninth People’s Hospital, Shanghai Jiaotong University School of Medicine, Shanghai, 200011 China; 5 Key Laboratory of Precision Diagnosis and Treatment for Hepatobiliary and Pancreatic Tumor of Zhejiang Province, Hangzhou, Zhejiang 310009 China; 6 Research Center of Diagnosis and Treatment Technology for Hepatocellular Carcinoma of Zhejiang Province, Hangzhou, Zhejiang 310009 China; 7 Clinical Medicine Innovation Center of Precision Diagnosis and Treatment for Hepatobiliary and Pancreatic Disease of Zhejiang University, Hangzhou, Zhejiang 310009 China; 8 Clinical Research Center of Hepatobiliary and Pancreatic Diseases of Zhejiang Province, Hangzhou, Zhejiang 310009 China; 9 Cancer Center, Zhejiang University, Hangzhou, Zhejiang 310009 China

**Keywords:** Slit2, Restenosis, Hif-1α, Smooth muscle cell, Bypass graft

## Abstract

Vascular smooth muscle cells (VSMCs) are involved in restenosis of bypass grafts and cause artery graft occlusion. This study aimed to explore the role of Slit2 in phenotypic switching of VSMCs and its effect on restenosis of vascular conduits. An animal model of vascular graft restenosis (VGR) was produced in SD rats and assessed by echocardiography. The expression of Slit2 and Hif-1α was measured in vivo and in vitro. After Slit2 overexpression, the migration and proliferation of VSMCs were detected in vitro, and the restenosis rates and phenotype of VSMCs were tested in vivo. The arteries of the VGR model presented significant stenosis, and Slit2 was decreased in VSMCs of the VGR model. In vitro, Slit2 overexpression inhibited the migration and proliferation of VSMCs, but Slit2 knockdown promoted migration and proliferation. Hypoxia induced Hif-1α but reduced Slit2, and Hif-1α negatively regulated Slit2 expression. Moreover, Slit2 overexpression weakened the rate of VGR and maintained the patency of artery bypass grafts, which suppressed the phenotypic switching of VSMCs. Slit2 inhibited the synthetic phenotype transformation to inhibit the migration and proliferation of VSMCs and delayed the VGR via Hif-1α.

**Supplementary Information:**

The online version contains supplementary material available at 10.1007/s12265-023-10384-8.

## Introduction

Coronary artery bypass graft surgery (CABG) is an efficient therapy that has been widely used to improve the outcome of patients with coronary artery disease [1]. The great saphenous vein, internal thoracic artery, radial artery, and right gastroepiploic artery are common bypass grafts utilized in CABG. However, neointima formation induces restenosis of bypass grafts (the patency rate ≥ 10 years: great saphenous vein is 50–60%, internal thoracic artery is 85–95%, radial artery is 89–91%, and right gastroepiploic artery is 62%), causing significant blood flow limitation in the revascularized region of the heart, which leads to poor outcomes for patients [2]. Moreover, the proximal and distal anastomoses of bypass grafts exhibit a high incidence of restenosis, which is caused by pathological activation of vascular smooth muscle cells (VSMCs) [3]. Under normal physiological conditions, VSMCs are exhibited hardly proliferation and migration, which present contractile phenotype and expresses characteristic proteins, such as α-smooth muscle actin (α-SMA) and myosin heavy chain 11 (MYH11) [4]. However, a number of previous studies have reported the mechanisms of SMCs participating in restenosis of bypass grafts, which is summarized as phenotype switching for contractile phenotype (α-SMA or MYH11 decreased) transformation into a synthetic phenotype (OPN or MGP as marker protein increased) and activated proliferation and migration [5, 6]. Meanwhile, VSMCs participating in anastomotic stenosis also led to graft restenosis, such as artery stenosis in the internal thoracic artery and hepatic artery anastomosis [7]. In recent research, Slit2, as a ligand of Robo receptors, has been shown to participate in axon guidance formation [8], and Slit2 inhibits migration and proliferation in rat airway smooth muscle cells [9]. However, Slit2 whether effect on phenotypic switching of VSMCs in vascular conduits is not clear.

However, some risk factors cause pathological changes in restenosis of bypass grafts, such as systemic biological factors (e.g., diabetes [10], hypercholesterolemia [11]) and other biological factors (e.g., inflammation [12], oxidative stress [13]), but the local microenvironment of vascular conduits (e.g., hypoxia [14]) can also participate in this process. The intima of the vessel presents hypoxic conditions triggering strong expression of hypoxia-inducible factor-1α (Hif-1α) and binding to a core DNA motif for transcription of genes, leading to neointimal formation [14, 15]. In this context, a recent study suggested that Slit2 suppresses hypoxia-induced inflammation and damage [16, 17] and that Slit2 regulates Hif-1α expression and activation [18]. However, the relationship between Slit2 and Hif-1α is not clear.

In this study, an animal model of artery bypass grafts was constructed to explore the role of Slit2 in restenosis of bypass grafts, and the relationship of Hif-1α with Slit2 was also detected in vivo and in vitro.

## Methods

### Reagents and Antibodies

Primary antibodies against Slit2 (Cat#ab7665), β-actin (Cat#ab8226), Hif-1α (Cat#ab1), MYH11 (Cat#ab82541), OPN (Cat#ab63856), VEGF(Cat#ab46154), total AKT (Cat#ab8805) and phosphorylation AKT (Cat#ab8933), and secondary goat anti-rabbit (Alexa Fluor® 488) (Cat#ab150077), donkey anti-mouse (Alexa Fluor® 555) (Cat#ab150106), donkey anti-goat (Alexa Fluor® 647) (Cat#ab150135), anti-rabbit (HRP) (Cat#ab288151), and anti-mouse (HRP) (Cat#ab97040) antibodies, as well as a CCK8 kit (Cat#ab228554), BrdU kit (Cat#ab287841), DAPI staining kit (Cat#ab104139), Hif-1α agonist (Cat#ab287115), and Hif-1α inhibitor (Cat#ab144422), were obtained from Cell Biolabs Abcam (Abcam, UK). The primary antibodies against αSMA (Cat# NB300-978) and MGP (Cat# H00004256-D01P) and the goat secondary antibody (HRP) (Cat# NB7357) were purchased from Novus (USA). A Transwell kit (Cat# ECM508, Sigma-Aldrich, USA) was used to measure the migration of cells. Fetal bovine serum (FBS, Cat# 0500, ScienCell, USA), 1:1 DMEM:F12 culture medium (Cat# 11330057, Gibco BRL, USA), penicillin–streptomycin (Cat# 15140122, Gibco BRL), and antibiotic–antimycotic solution (Cat# 15240062, Gibco BRL) were used to culture cells. Shanghai Genechem Company produced adeno-associated virus (AAV) for Slit2 overexpression (AAV-Slit2( +)) and the blank control (AAV-blank). The negative and Slit2 siRNA (GTACACACACACGTTCG) for mice was purchased from the Genomeditech Company (Shanghai. China). The 8–0 Prolene suture was used in animal experiments (Ethicon, Shanghai, China). BCA Protein Assay Kits (Cat #P0010S) were purchased from Beyotime (China). The Immobilon™ Western chemiluminescent HRP substrate was used for western blotting (Cat# WBKLS0500, Millipore, USA).

### Animals and Experiments

The animal study protocols were approved by the Animal Care and Use Committee of the Second Affiliated Hospital, Zhejiang University School of Medicine, China, which conforms to the guidelines from Directive 2010/63/EU of the European Parliament regarding the protection of animals used for scientific purposes.

Male SPF-grade Sprague–Dawley (SD) rats were purchased from Charles River Company (China). The SD rats were allowed to adapt to the environment for 1 week prior to experimentation in the animal house of the Second Affiliated Hospital, Zhejiang University School of Medicine. All animal experiments followed the Replacement, Refinement, and Reduction (3R) principle [19].

For construction of an animal model of artery bypass grafting, 12-week-old SD rats underwent abdominal aorta (average vascular diameter was 2.2 ± 0.2 mm) transplantation by two end-side anastomoses. The vascular conduits of donor were gathered from the homologous paternal origin of transplant recipient (Supplementary Fig. S1). The process of artery bypass grafting operation followed the following steps: (1) pentobarbital sodium was administered to the rats at 30 mg/kg via intraperitoneal injection. (2) Median laparotomy was adopted to expose the abdominal aorta by pushing away the bowel and other tissues. (3) For systemic heparinization, 100 IU/kg heparin was administered to the inferior vena cava. (4) Through end-to-side continuous suturing, the donated vessel (SD aorta) was implanted on the infrarenal aorta with 8–0 prolene sutures. (5) Later, 3–0 silk sutures were adopted to ligate the native aorta between the two anastomoses to avoid shunting (Supplementary Fig. 1).

The patency of aorta bypass grafts was assessed by a Vevo 2100 instrument (FujiFilm Visual Sonics) equipped with an MS-250 imaging transducer, which included vascular diameter (mm) and pulsed wave (PW) Doppler mean velocity (mm/s), when the operation was completed and 16 weeks after the operation. Each SD rat was assessed differently 3 times, and the data was gathered. Echocardiography (echo) and data analysis were performed by an independent observer who did not participate in the operation. For collection of the aorta bypass grafts of rats, the rats were euthanized by isoflurane inhalation and intraperitoneal injection of pentobarbital sodium (30 mg/kg body weight).

Twenty SD rats had undergone abdominal aorta transplantation, and following the echo measurement, the ratio of bypass graft patency < 50% was identified as vascular graft restenosis (VGR), and > 90% was identified as non-VGR. The VGR and non-VGR groups contained 5 rats for each group.

Another group of SD rats (*n* = 20) underwent abdominal aorta transplantation and was randomly divided into two groups: (1) AAV-blank injection (*n* = 10) and (2) AAV-Slit2( +) injection (*n* = 10). The AAV were resuspended into saline, and the concentration was 5 × 10^13^ GC/mL. Each rat was injected with AAV at a concentration of 5 × 10^11^ GC/mL/100 μL via the tail vein.

### Immunofluorescence

The aorta bypass grafts were collected and fixed with 4% paraformaldehyde for 12 h and embedded at the optimal cutting temperature. The tissues were cut into frozen sections that were 6 μm thick and labeled with different antibodies: anti-Slit2 (1:50), anti-Hif-1α (1:50), and anti-αSMA (1:100). After incubation with Alexa 488- or Alexa 555- or Alexa 647-conjugated secondary antibodies (1:1000), all sections were detected on a machine after mounting with DAPI-containing mounting agent and exposed with a laser confocal microscope (Zeiss 880).

### Primary Smooth Muscle Culture and Treatment

The aortic media VSMCs in SD rats (12 weeks) were used for in vitro primary VSMC culture. The rats were euthanized by intraperitoneal injection of pentobarbital sodium (50 mg/kg), and the aorta explants were removed. Then, the vessel was cut open, and the lining of the vessel was scraped off to remove endothelial cells. The tissues were cut into 4 mm^2^ sections, blocked on culture dishes, and cultured in F12:DMEM (1:1) with 20% FBS containing 1 × penicillin–streptomycin. After the cells migrated out from the edge of the tissue for approximately 5 or 7 days, the cells were transferred to new medium for 2 days to collect primary cells for experiments.

The primary VSMCs were transformed into 60-mm culture dishes, and cells at 70–80% confluence underwent different treatments. A total of 1 × 10^5^ GC/mL AAV-Slit2( +) or AAV-blank was added to the culture medium. After AAV infection for 24 h, the efficiency of transfection and the expression of some genes were tested by western blotting, and the migration and proliferation were observed by a kit. (1) VSMCs were incubated with hypoxia and 2% O_2_ for different times (1, 3, 6, and 9 h), and the expression of Slit2 and Hif-1α was measured. Two VSMCs were stimulated with 2 μM Hif-1α agonist under normoxia or 20 nM Hif-1α inhibitor under hypoxia, and the concentration of Slit2 was detected.

### Hypoxia Treatment

The cells were cultured into 60-mm culture dishes and then put into a hypoxic incubator (Forma Steri-Cycle i250, Thermo Fisher, USA) for hypoxia treatment. The hypoxic incubator perfused nitrogen to eliminate content of oxygen (final O_2_ = 2%).

### Oligo Transfection

SiRNA of Slit2 or NC was performed using Lipofectamine iMAX reagent according to the manufacturer’s protocol.

### Western Blot Analysis

The proteins were lysed from tissues or cells, and concentrations were detected by a BCA kit as previously described [20]. The total protein (20 µg/sample) was loaded and separated on a 10% SDS–polyacrylamide gel. Then, the proteins were transferred onto a PVDF membrane and incubated with Slit2 (1:1000), Hif-1α (1:1000), αSMA (1:2000), MYH11 (1:1000), OPN (1:1000), MGP (1:1000), and β-actin (1:2000) antibodies overnight at 4 °C. Following a secondary HRP-conjugated goat anti-rabbit IgG (1:5000), goat anti-mouse IgG (1:5000), or donkey anti-goat IgG (1:5000) incubation for 2 h at room temperature, the membrane was washed and exposed by using an ECL kit. β-Actin was used as an internal control.

### Transwell Assay

After VSMCs were transfected with AAV-blank and AAV-Slit2( +) for 24 h, the cells were starved overnight in serum-free medium. The mixture of cells was added to the upper chamber, and culture medium with 10% FBS was added to the bottom chamber. The chambers were incubated for 12 h, and the chambers were removed from the medium and scraped to remove the nonmigrated cells from the upper surface. The migrating cells were stained with labeling buffer to take pictures with a microscope (Olympus, Japan), dye was eluted from the cells, and a microplate reader (Bio-Rad, USA) was used at OD = 560 nm to assess migrating cells.

### Wound-Healing Assay

VSMCs were seeded in 6-well plates and transfected with AAV-blank and AAV-Slit2( +) for 24 h. The cells were starved overnight in serum-free medium. The wound scratch was made with a 200 μL tip, and the culture medium was refreshed. Images were taken by a microscope (Olympus, Japan) at time points of 0 and 12 h. The velocity (μm/min) was calculated with Image-Pro Plus version 6.2 (Media Cybernetics, USA).

### CCK8 Assay

VSMCs were seeded in 96-well plates and transfected with AAV-blank and AAV-Slit2( +) for 24 h. Then, CCK8 staining was added to each well, incubated for 2 h, and quantified by a microplate reader (Bio-Rad, USA) at OD = 450 nm to assess proliferating cells.

### BrdU Assay

VSMCs were seeded in 96-well plates and transfected with AAV-blank and AAV-Slit2( +) for 24 h, and 10 µM BrdU was added to each well for 4 h. The cells were fixed and incubated with anti-BrdU for 1 h, HRP-conjugated secondary antibody for 30 min, and reacted with TMB substrate for 15 min. After stopping the reaction, the OD values were measured at 450–540 nM within 30 min.

### Statistical Analysis

The results are shown as the mean ± SD. Student’s *t* test was used to compare two means. Dunnett’s multiple comparison test was used for one-way ANOVA to compare > 2 groups. Repeated measures ANOVA performed by Dunnett’s multiple comparison test was used to study the changes in the average score at ≥ 3 time points. *P* < 0.05 was considered statistically significant. Statistical analysis was performed using GraphPad Prism 7.0 (GraphPad Prism software) and SPSS 14.0 (IBM Corp) for Windows.

## Results

### Slit2 Decreased in Restenosis of Vascular Conduits in a Rat Model

**Fig. 1:**
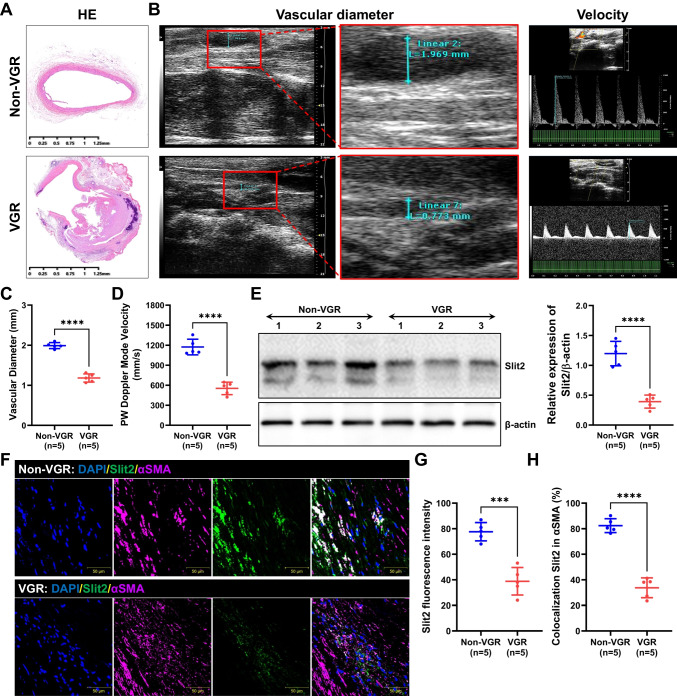
The expression and location of Slit2 in vascular graft restenosis in a rat model. The SD rats underwent abdominal aortic transplantation, and the VGR and non-VGR arteries were collected to measure the expression and location of Slit2. **A** The HE staining of VGR and non-VGR arteries. **B** Images of echocardiography. **C**–**D** The vascular diameter and PW velocity were measured (*n* = 5, ****P < 0.0001 with Student’s *t* test). **E** Western blotting was used to detect the level of Slit2 (β-actin was used as an internal control, *n* = 5, ****P < 0.0001 with Student’s *t* test). **F** Staining for DAPI (blue), Slit2 (green), and αSMA (pink) was performed. **G** The fluorescence intensity of Slit2 (*n* = 5, ***P < 0.001 with Student’s *t* test). **H** The ratio of colocalization of Slit2 in α-SMA (*n* = 5, ****P < 0.0001 with Student’s *t* test)

To verify the expression of Slit2 in vascular graft restenosis, we generated a rat model of artery bypass grafting. The immunohistochemistry showed lumen stenosis in VGR group (Fig. 1A). Following echocardiography, the VGR group exhibited significant restenosis compared with the non-VGR group (vascular diameter: VGR = 1.99 ± 0.08 mm vs. non-VGR = 1.18 ± 0.10 mm, *P* < 0.01; PW velocity: VGR = 1173.00 ± 117.50 mm/s vs. non-VGR = 552.90 ± 93.35 mm/s, *P* < 0.01) (Fig. 1B–D). The results of western blotting showed that the expression of Slit2 significantly decreased almost in the VGR (almost 3.05-fold decrease, *P* < 0.01) (Fig. 1E). To further validate the expression and location of Slit2 in the vascular graft, we labeled Slit2 and αSMA (marker of smooth muscle cells) on vascular conduits of the VGR and non-VGR groups. The immunofluorescence data showed that Slit2 exhibited stronger expression (VGR = 38.80 ± 10.76 vs. non-VGR = 77.60 ± 7.16, *P* < 0.01) (Fig. 1F and G) in αSMA-positive cells (VGR = 33.70 ± 7.79% vs. non-VGR = 82.39 ± 5.44%, *P* < 0.01) (Fig. 1F and H) in the non-VGR group compared with the VGR group, which also indicated that Slit2 was located on VSMCs.

### Slit2 Regulated the Migration and Proliferation of Smooth Muscle Cells

**Fig. 2:**
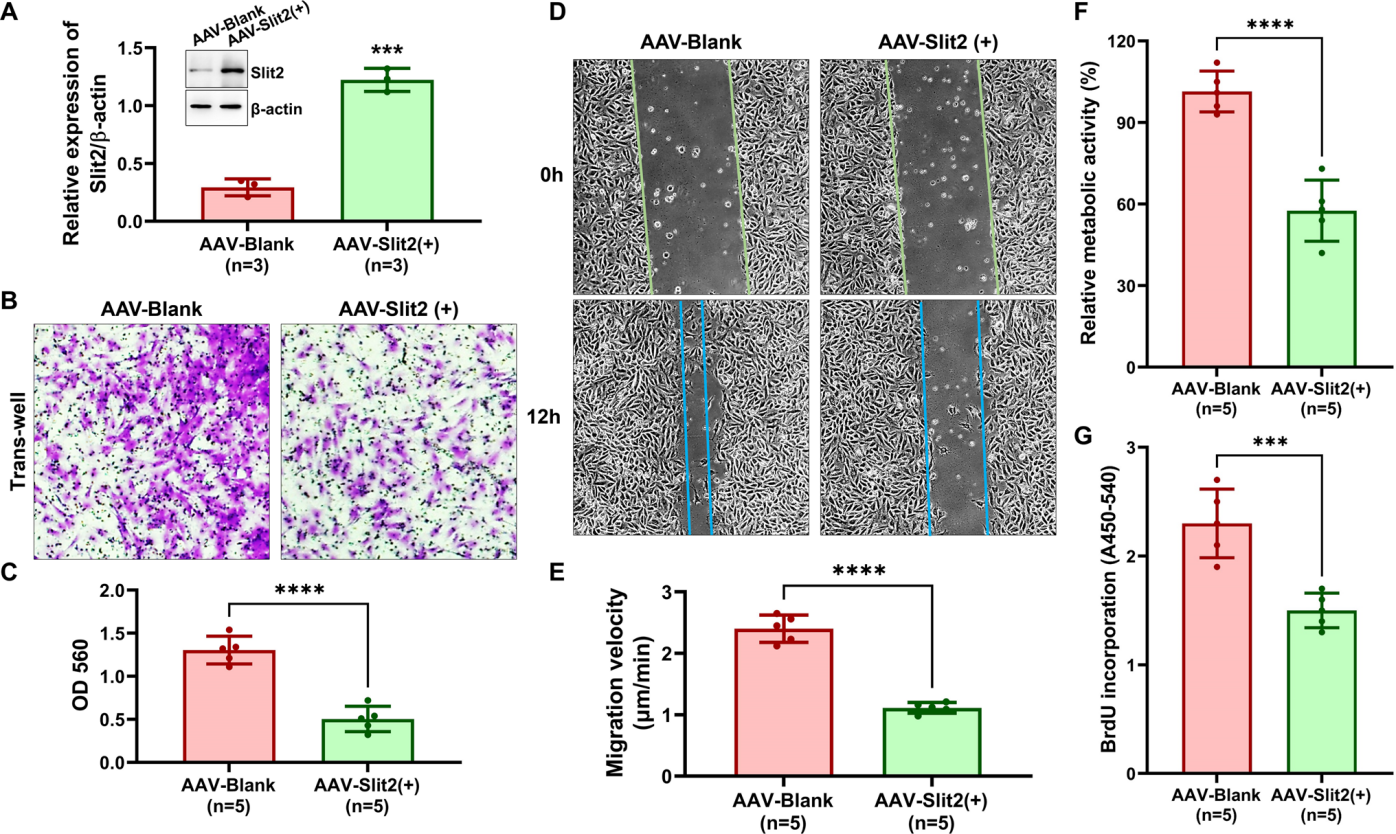
Slit2 overexpression regulated the migration and proliferation of VSMCs. AAV-blank and AAV-Slit2( +) were transfected into VSMCs, and migration was detected by Transwell and wound-healing assays. Proliferation was measured by CCK8 and BrdU assays. **A** Western blotting was used to detect the efficiency of Slit2 overexpression with AAV-Slit2 ( +) transfected into VSMCs (β-actin was used as an internal control, *n* = 3, ***P < 0.001 with Student’s *t* test). **B**–**C** The effect of Slit2 overexpression on VSMC migration by Transwell assays (*n* = 5, ***P < 0.001 with Student’s *t* test). **D**–**E** Wound healing was used to test the migration velocity of VSMCs (*n* = 5, ***P < 0.001 with Student’s *t* test). **F**–**G** The proliferation of VSMCs was detected by CCK8 or BrdU assays (*n* = 5, ***P < 0.001, ****P < 0.0001 with Student’s *t* test)

The migration and proliferation of VSMCs are important cytological events of restenosis of vascular bypass grafts [21, 22]. Thus, we used AAV infected with VSMCs to overexpress Slit2 (the overexpression efficiency is shown in Fig. 2A) to determine whether Slit2 regulated the migration (tested by Transwell and wound-healing assays) and proliferation (tested by CCK8 and BrdU assays) of VSMCs. Compared with AAV-blank transfection, Slit2 overexpression significantly inhibited migration (Transwell: AAV-blank = 2.40 ± 0.22 vs. AAV-Slit2( +) = 1.11 ± 0.09, *P* < 0.01 (Fig. 2B and C); wound healing: AAV-blank = 1.30 ± 0.16 μm/min vs. AAV-Slit2( +) = 0.50 ± 0.15 μm/min, *P* < 0.01 (Fig. 2D and E)) and proliferation (CCK8: AAV-blank = 101.4 ± 7.50% vs. AAV-Slit2( +) = 57.60 ± 11.24%, *P* < 0.01 (Fig. 2F); BrdU: AAV-blank = 2.30 ± 0.32 vs. AAV-Slit2( +) = 1.50 ± 0.16, *P* < 0.01 (Fig. 2G)) of VSMCs.

**Fig. 3:**
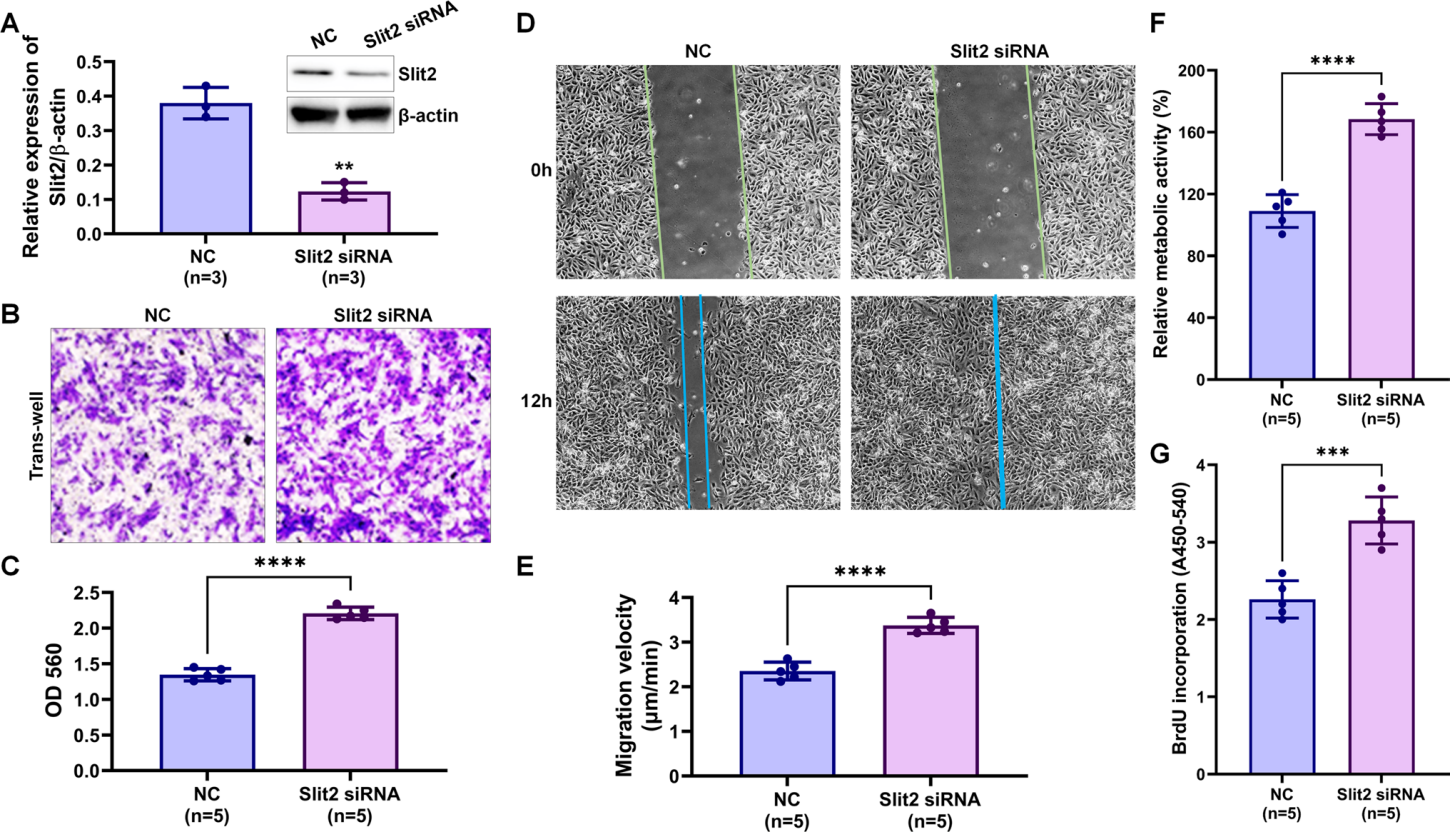
Slit2 knockdown regulated the migration and proliferation of VSMCs. Negative control (NC) and Slit2 siRNA were transfected into VSMCs, and migration was detected by Transwell and wound-healing assays. Proliferation was measured by CCK8 and BrdU assays. **A** Western blotting was used to detect the efficiency of Slit2 knockdown with Slit2 siRNA transfected into VSMCs (β-actin was used as an internal control, *n* = 3, **P < 0.01 with Student’s *t* test). **B**–**C** The effect of Slit2 knockdown on VSMC migration by Transwell assays (*n* = 5, ****P < 0.0001 with Student’s *t* test). **D**–**E** Wound healing was used to test the migration velocity of VSMCs (*n* = 5, ****P < 0.0001 with Student’s *t* test). **F**–**G** The proliferation of VSMCs was detected by CCK8 or BrdU assays (*n* = 5, ***P < 0.001, ****P < 0.0001 with Student’s *t* test)

To further verify the effect of Slit2 on VSMCs, a specific siRNA of Slit2 was transfected into VSMCs to knockdown the expression of Slit2 and observed migration and proliferation of VSMCs (the knockdown efficiency is shown in Fig. 3A). Compared with negative siRNA (NC) transfection, Slit2 siRNA significantly promoted migration (Transwell: NC = 1.35 ± 0.09 vs. Slit2 siRNA = 2.21 ± 0.09, *P* < 0.01 (Fig. 3B and C); wound healing: NC = 2.35 ± 0.20 μm/min vs. Slit2 siRNA = 3.38 ± 0.18 μm/min, *P* < 0.01 (Fig. 3D and E)) and proliferation (CCK8: NC = 109.0 ± 10.61% vs. Slit2 siRNA = 168.4 ± 10.09%, *P* < 0.01 (Fig. 3F); BrdU: NC = 2.26 ± 0.24 vs. Slit2 siRNA = 3.28 ± 0.30, *P* < 0.01 (Fig. 3G)) of VSMCs.

### Hypoxia Reduced Slit2 Expression via Hif-1α

**Fig. 4:**
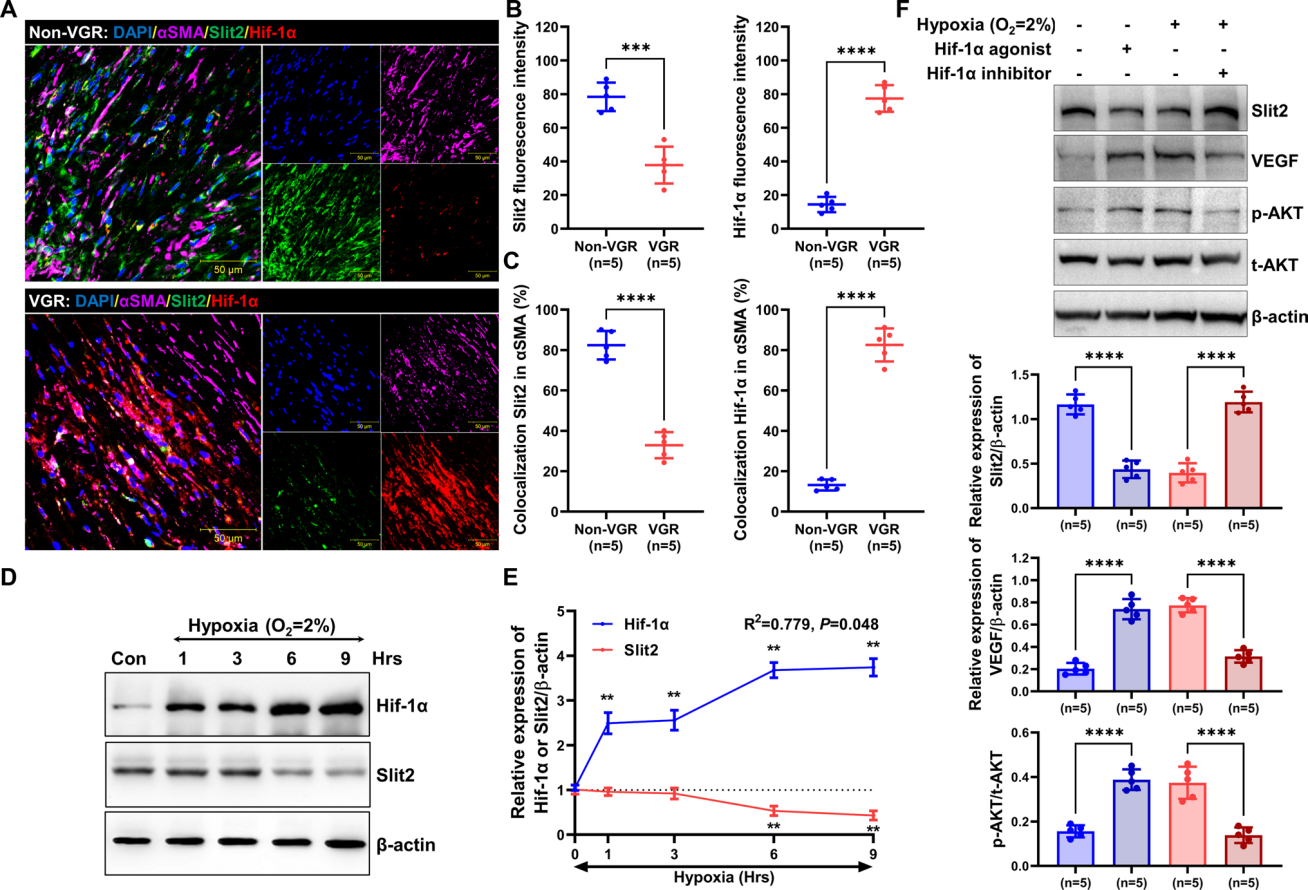
Hypoxia downregulated Slit2 expression via Hif-1α. **A** DAPI (blue), Slit2 (green), Hif-1α (red), and αSMA (pink) were labeled on the arteries of the VGR and non-VGR groups. **B** The fluorescence intensity of Slit2 and Hif-1α (*n* = 5, ***P < 0.001, ****P < 0.0001 with Student’s *t* test). **C** The ratio of colocalization of Slit2 and Hif-1α in α-SMA (*n* = 5, ****P < 0.0001 with Student’s *t* test). **D** and **E** VSMCs were incubated with hypoxia for 1, 3, 6, and 9 h, and the expression of Slit2 and Hif-1α was detected by western blotting. β-actin was used as an internal control (*n* = 5, **P < 0.01 with Dunnett’s multiple comparison test). **F** VSMCs were treated with agonist or inhibitor under normoxia or hypoxia, and the expression was tested by western blotting (β-actin was used as an internal control, *n* = 5, ****P < 0.0001 with one-way ANOVA)

The local microenvironment of hypoxia induces Hif-1α expression and leads to neointimal formation [14, 15]. In this context, the levels of Hif-1α and Slit2 were detected in vascular bypass grafts of rats with VGR. After labeling of Hif-1α, Slit2, and αSMA, Hif-1α exhibited strong expression (expression: VGR = 77.40 ± 7.89 vs. non-VGR = 14.40 ± 4.51, *P* < 0.01; colocalization: VGR = 82.54 ± 8.20% vs. non-VGR = 13.18 ± 2.72%, *P* < 0.01), but Slit2 presented weak expression (expression: VGR = 37.80 ± 10.89 vs. non-VGR = 78.40 ± 8.47, *P* < 0.01; colocalization: VGR = 32.88 ± 6.48% vs. non-VGR = 82.42 ± 7.06%, *P* < 0.01) in the region of αSMA-positive VGR (Fig. 4A–C), which suggested that Slit2 was related to Hif-1α. Furthermore, the VSMCs were incubated with hypoxia for different times (1, 3, 6, and 9 h), and hypoxia caused a negative relationship between the expression of Hif-1α and Slit2 (*R*^2^ = 0.779, *P* = 0.048), which also occurred in a time-dependent manner (Fig. 4D and E). To further identify the association of Hif-1α with Slit2, VEGF, and phosphorylation of AKT, we treated VSMCs with an agonist and inhibitor of Hif-1α under normoxia or hypoxia, and the results showed that activating Hif-1α significantly inhibited Slit2 expression but upregulated VEGF and activation of AKT under normoxia. However, blocking Hif-1α significantly promoted Slit2 levels but downregulated VEGF and activation of AKT under hypoxia in VSMCs (*P* < 0.01) (Fig. 4F).

### Slit2 Reduced VGR and Phenotypic Switching of VSMCs in Rats

**Fig. 5:**
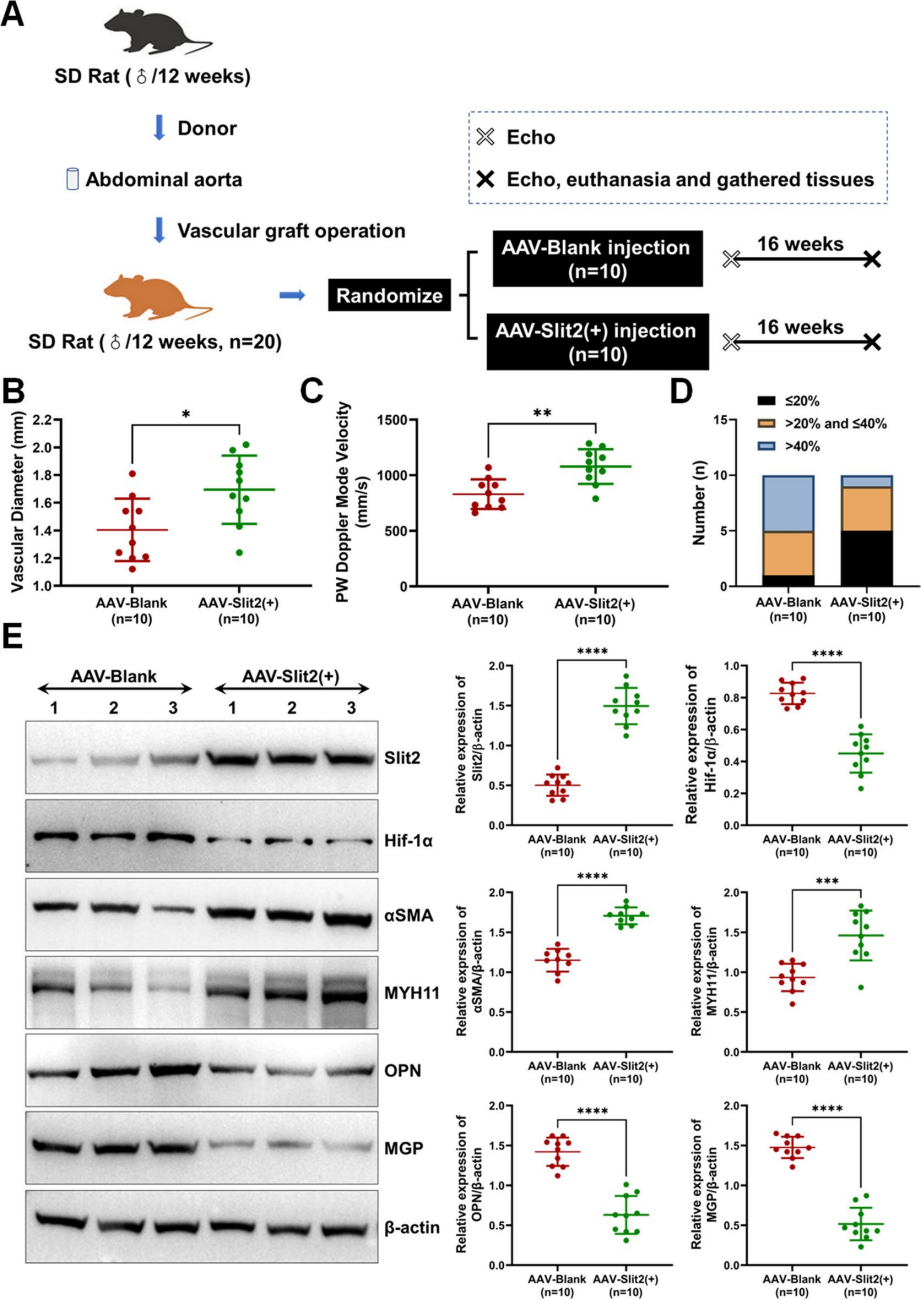
Overexpression of Slit2 delayed VGR and phenotypic switching of VSMCs in rats. AAV-blank and AAV-Slit2( +) were injected into the rats with abdominal aortic transplantation. The patency of aorta bypass grafts was observed by echocardiography, and phenotype switching of VSMCs was detected by western blotting. **A** Flowchart of the animal experiments. **B** and** C** The vascular diameter and PW velocity were measured (*n* = 10, *P < 0.05, **P < 0.01 with Student’s *t* test). **D** The number of different stenosis rates in the rats with abdominal aortic transplantation. **E** The Slit2, Hif-1α, contractile phenotype (αSMA and MYH11), and synthetic phenotype (OPN and MGP) were tested by western blots (β-actin was used as an internal control, *n* = 10, ***P < 0.001, ****P < 0.0001 with Student’s *t* test)

As Slit2 inhibited the migration and proliferation of VSMCs in vitro, AAV-blank or AAV-Slit2( +) was injected into the rats with aortic transplantation to verify the effect of Slit2 on VGR (the animal study flowchart is shown in Fig. 5A). The echocardiography showed that Slit2 overexpression delayed the decline in vascular graft diameter (AAV-blank = 1.40 ± 0.23 mm vs. AAV-Slit2( +) = 1.69 ± 0.25 mm, *P* < 0.05) (Fig. 5B) and PW velocity (AAV-blank = 829.60 ± 133.30 mm/s vs. AAV-Slit2( +) = 1078.00 ± 156.60 mm/s, *P* < 0.05) (Fig. 5C) and decreased the levels of severe stenosis (ratio of stenosis > 40%: AAV-blank = 5 vs. AAV-Slit2( +) = 1; ratio of stenosis > 20% and ≤ 40%: AAV-blank = 4 vs. AAV-Slit2( +) = 4; ratio of stenosis ≤ 20% AAV-blank = 1 vs. AAV-Slit2( +) = 5) (Fig. 5D). Phenotypic switching is an important mechanism leading to pathological changes in VSMCs in the VGR, such as decreased contractile phenotype molecules (αSMA and MYH11) and increased synthetic phenotype molecules (OPN and MGP) [5, 6]. Thus, vascular conduits from AAV-blank- or AAV-Slit2( +)-injected rats with aortic transplantation were used to culture primary VSMCs, and the expression of contractile phenotype and synthetic phenotype molecules was measured. The results showed that Slit2 overexpression significantly promoted the expression of αSMA and MYH11 but significantly inhibited OPN and MGP (*P* < 0.01) (Fig. 5E); meanwhile, Slit2 overexpression also suppressed Hif-1α (*P* < 0.01) (Fig. 5E).

## Discussion

Collectively, the results of this study support Slit2 as a novel factor that participates in restenosis of aortic bypass grafts by reducing the pathological phenotypic switch of VSMCs, and hypoxia decreases Slit2 expression. More specifically, our data showed that Slit2 was decreased on aortic bypass grafts. Moreover, Slit2 overexpression suppressed the migration and proliferation of VSMCs in vitro and reduced the levels of stenosis in vivo. Our data identify a potential protective factor that antagonizes VGR.

VGR was a key risk factor for the incidence of graft failure, even though arterial grafts exhibited higher patency rates than saphenous vein grafts [2]. Neointima formation, as an important pathological hallmark of VGR, leads to almost 50% of grafts failing within 10 years, and VSMCs, as the major cell type of grafts, are involved in the whole process [1, 23]. The characteristics of VSMCs suggest that the different roles are involved in different functions: (1) in healthy vessels, contractile or differentiated VSMCs express contractile cytoskeletal proteins and show extremely low proliferation and migration to contract and relax to regulate vessel tone and blood pressure [24, 25]. (2) Under vascular injury conditions, VSMCs switch from the quiescent contractile differentiated phenotype to a dedifferentiated synthetic phenotype and show strong proliferation and migration to repair the vasculature, causing thickening of the intima [5, 26]. These characteristics of VSMCs are called “phenotype switching,” which is a protective vascular mechanism under physiological conditions. However, risk factors caused constant phenotypic switching of VSMCs and triggered severe neointima formation, which led to vascular occlusion and decreased patency, especially for VGR. Previous studies have reported that both vein and arterial grafts exhibit localization hypoxia at the anastomosis [27] and arteriovenous fistulas [28], which regulate the migration and proliferation of VSMCs to promote neointima formation [29]. Moreover, hypoxia induced Hif-1α as a hypoxia-response element that increased sharply and bound a core DNA motif to regulate the expression of hypoxia-responsive genes, such as stromal cell-derived factor and thrombospondin-1, to increase the proliferation and migration of VSMCs [15, 30]. However, low expression of Hif-1α reduced neointimal formation after vascular injury [15]. Thus, Hif-1α, as a hypoxia-induced risk factor, induced VSMC phenotype switching and promoted neointimal formation to cause VGR. In our study, Slit2 was decreased in VSMCs of VGR, but Hif-1α was increased, and Hif-1α negatively regulated the expression of Slit2. The previous studies showed that Hif-1α induced AKT activation and VEGF expression, and VEGF signaling pathway activation promoted migration and proliferation of VSMCs [31, 32]. All of these data hint that Hif-1α might regulate Slit2 via VEGF-AKT pathway. Furthermore, Slit2 overexpression inhibited VSMC migration and proliferation and weakened restenosis of vascular conduits and the synthetic phenotype change of VSMCs in rats with aortic transplantation. Therefore, our data indicated that Slit2 resisted VGR by inhibiting VSMC phenotype switching, but Hif-1α antagonized Slit2 expression to decrease the protective effect of Slit2.

Slit2 belongs to the Slit family, which contains 4 tandem leucine-rich repeats (LRRs), 7 epidermal growth factor (EGF)-like repeats, an agrin–laminin–perlecan–slit (ALPS)-conserved spacer motif, and a cystine knot [33]. The physiological function of Slit2 is to form axon guidance molecules by binding Robo receptors [8, 34]. Previous studies reported that Slit2 inhibited the migration of VSMCs by blocking the activation of the small GTPase Rac1 [35], and the small GTPase Rac1 participated in cytoskeletal synthesis [36]. Moreover, the phenotypic switching of VSMCs involves cytoskeletal rearrangement with decreased contractile cytoskeletal proteins (αSMA and MYH11) [6]. These data suggested that Slit2 participates in cytoskeletal rearrangement to regulate phenotype switching of VSMCs. Our data supported this hypothesis; overexpression of Slit2 promoted the contractile cytoskeletal proteins αSMA and MYH11 and inhibited the migration and proliferation of VSMCs. In the present study, we also found that Slit2 decreased in the vasculature of VGR and was downregulated by Hif-1α. However, Slit2 was abundantly expressed in various aortas [35], which suggested that Slit2 may be a regulatory factor that maintains vascular homeostasis and patency, but Hif-1α attenuated the function of Slit2 by suppressing expression in aortic bypass grafts. Further studies demonstrated that Slit2 regulated Hif-1α expression to affect thyroid cancer cells [18]; however, inhibition of miR-200b-3p targeting and downregulation of Slit2 improved hypoxia-ischemic brain damage [17]. All of these results indicated that Slit2 delayed hypoxia-induced restenosis on aorta bypass grafts via inhibition of Hif-1α. Eventually, Slit2 and Hif-1α present bidirectional regulation in VSMCs of grafts.

## Conclusion

In this study, Slit2 decreased in the rats with VGR, but overexpression of FSTL1 inhibited migration and proliferation of VSMCs in vitro and reversed restenosis of aorta bypass grafts and the synthetic phenotype of VSMCs in vitro. Moreover, Hif-1α reduced the expression of Slit2, which is a novel mechanism of Slit2 in VSMCs.

### Supplementary Information

Below is the link to the electronic supplementary material.

Supplementary Figure 1. The schematic diagram of vascular graft operation. (PNG 48 kb)

High resolution image (TIF 110 kb)

## Data Availability

Data supporting the findings of this study are available within the article and its supplementary materials.
