# Supplementary figures and images for: Hif-1α/Slit2 Mediates Vascular Smooth Muscle Cell Phenotypic Changes in Restenosis of Bypass Grafts
Source: J Cardiovasc Transl Res. 2023 Apr 25;16(5):1021–31. doi: 10.1007/s12265-023-10384-8 (PMC10615989; doi:10.1007/s12265-023-10384-8)

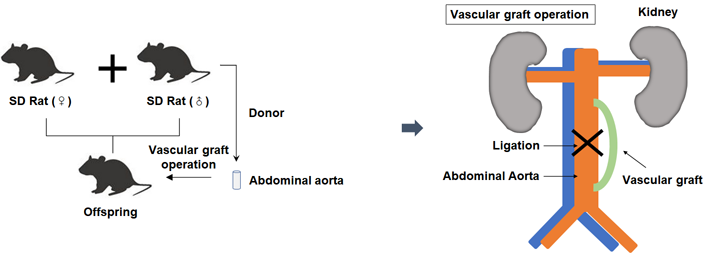

Supplement: Supplementary file 1 — The schematic diagram of vascular graft operation. (PNG 48 kb) [file 12265_2023_10384_Fig6_ESM.png]

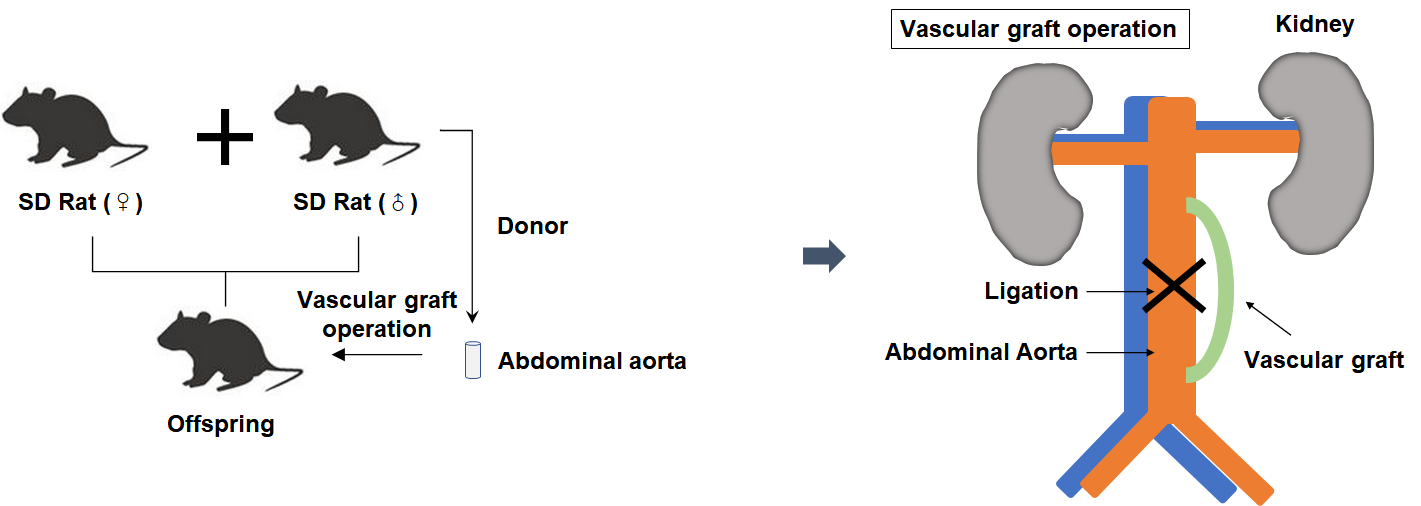

Supplement: Supplementary file 2 — High resolution image (TIF 110 kb) [file 12265_2023_10384_MOESM1_ESM.tif]
